# Surface display of heterologous proteins in *Bacillus thuringiensis *using a peptidoglycan hydrolase anchor

**DOI:** 10.1186/1475-2859-8-48

**Published:** 2009-09-16

**Authors:** Xiaohu Shao, Mengtian Jiang, Ziniu Yu, Hao Cai, Lin Li

**Affiliations:** 1 State Key Laboratory of Agricultural Microbiology, Huazhong Agricultural University, Wuhan 430070, China; 2 College of Life Science and Technology, Huazhong Agricultural University, Wuhan 430070, China

## Abstract

**Background:**

Previous studies have revealed that the lysin motif (LysM) domains of bacterial cell wall-degrading enzymes are able to bind to peptidoglycan moieties of the cell wall. This suggests an approach for a cell surface display system in Gram-positive bacteria using a LysM-containing protein as the anchoring motif. In this study, we developed a new surface display system in *B. thuringiensis *using a LysM-containing peptidoglycan hydrolase, endo-*β*-*N*-acetylglucosaminidase (Mbg), as the anchor protein.

**Results:**

Homology searching in the *B. thuringiensis *YBT-1520 genome revealed a putative peptidoglycan hydrolase gene. The encoded protein, Mbg, exhibited substantial cell-wall binding capacity. The deduced amino acid sequence of Mbg was structurally distinguished as an N-terminal domain with two tandemly aligned LysMs and a C-terminal catalytic domain. A GFP-fusion protein was expressed and used to verify the surface localization by Western blot, flow cytometry, protease accessibility, SDS sensitivity, immunofluorescence, and electron microscopy assays. Low-level constitutive expression of Mbg was elevated by introducing a sporulation-independent promoter of *cry3Aa*. Truncated Mbg domains with separate N-terminus (Mbgn), C-terminus (Mbgc), LysM_1_, or LysM_2 _were further compared for their cell-wall displaying efficiencies. The Mbgn moiety contributed to cell-wall anchoring, while LysM_1 _was the active domain. Two tandemly repeated Mbgns exhibited the highest display activity, while the activity of three repeated Mbgns was decreased. A heterologous bacterial multicopper oxidase (WlacD) was successfully displayed onto the surface of *B. thuringiensis *target cells using the optimum (Mbgn)_2 _anchor, without radically altering its catalytic activity.

**Conclusion:**

Mbg can be a functional anchor protein to target different heterologous proteins onto the surface of *B. thuringiensis *cells. Since the LysM domain appears to be universal in Gram-positive bacteria, the strategy presented here could be applicable in other bacteria for developing this type of system.

## Background

Gram-positive bacteria are a group of microorganisms that have rigid cell walls but lack outer membrane envelopes. This cell wall structure is particularly amenable to development of surface display strategies in terms of simplification of protein secretion [1]. One of these Gram-positive bacteria, the spore-forming bacterium *B. thuringiensis*, has been characterized by its ability to synthesize a large amount of parasporal crystal proteins [2]. This organism has been used as a successful biopesticide for more than 50 years and has been recognized as a uniquely safe, maneuverable, and cost-effective tool for pest control [3]. These features make this bacterium an attractive candidate for using of a cell surface display strategy to develop a multifunctional system, for example, a combined insecticidal and antimicrobial system, to extend its agricultural or biotechnological applications.

Previously studied *Bacillus *systems have almost exclusively used various cell surface bound proteins as their anchoring motifs. These have included proteins such as PrsA, CwlB and CwlC of *B. subtilis *[4,5], and the surface layer (S-layer) protein from *B. anthracis *[6]. However, study of the available cell wall anchoring proteins in *B. thuringiensis *is lacking. To date, only a few anchor proteins, such as S-layer proteins or spore coat proteins, have been exploited to immobilize heterologous proteins onto the surface of vegetative cells [7,8] or spores [9].

Bacterial peptidoglycan hydrolases are a group of endogenous autolysins that hydrolyze the glycosidic bonds in the peptidoglycan of their own cell walls [10-12]. These enzymes have been associated with the processing events that are required for cell-wall expansion, peptidoglycan turnover, daughter cell separation, and sporulation [12,13]. Typically, peptidoglycan hydrolases contain the tandemly repeated sequences known as the lysin motif (LysM) [14], which is able to bind to peptidoglycans of various Gram-positive bacteria [12]. These findings suggest an approach for a cell surface display system in Gram-positive bacteria, through the use of LysM-containing proteins as the anchors. In fact, AcmA, one of major autolysins from *L. lactis*, which contains three LysMs at its C-terminal moiety, has been exploited in this way for development of cell surface display systems [15,16].

Endo-*β*-*N*-acetylgucosaminidases (termed '*N*-acetylglucosaminidases' in brief) are a widespread group of bacterial hydrolases. They hydrolyze the glycosidic bond, i.e, GlcNAc *β*-1,4 GlcNAc of peptidoglycan, releasing the *N*-glycan moiety [17,18]. Several characteristics make *N*-acetylglucosaminidases a logical choice as a surface anchoring motif. Firstly, they bind directly to high-MW peptidoglycans using one or several LysM domains, whose presence greatly enhances the peptidoglycan-binding capacities. Secondly, they are stably expressed throughout the vegetative growth phase of the bacterium. Thirdly, most of these enzymes have common structures that can be distinguished as a binding domain and a catalytic domain, and the latter can be fairly easily deleted with straightforward genetic manipulations (for reviews, see [18,19]).

To date, no effort has yet been made to investigate the possibility of a cell surface display system for *B. thuringiensis *mediated by a peptidoglycan hydrolase anchor. In the present study, we report on an approach for displaying heterologous proteins on the cell surface of *B. thuringiensis*, using a putative peptidoglycan hydrolase (*N*-acetylglucosaminidase) as the anchoring motif. The surface localization of chimeric proteins was first confirmed in a modular system with green fluorescence protein (GFP) as the reporter. An optimum system was then subsequently obtained by comparing the efficiency of different binding domains. The system was further applied to display a previously characterized laccase from *Shigella dysenteriae *[20] onto the surface of *B. thuringiensis *target cells, and the resulting surface localization and whole-cell enzymatic activity was investigated.

## Results

### Identification of the putative peptidoglycan-binding protein genes in the *B. thuringiensis *YBT-1520 genome sequence

The 5.4 Mb genomic DNA of *B. thuringiensis *subsp. *kurstaki *wild-type strain YBT-1520 comprises over 5600 ORFs (data not published). A whole-genome analysis of the corresponding proteins in this strain was performed with the online tool "softberry" [21], using the genome data of *B. thuringiensis *subsp. *konukian *str 97-27 as the reference (GenBank: AE017355). Over 60 ORFs were suggested to be genes associated with cell wall hydrolysis. Three putative cell-wall binding proteins, designated as Mbg, Mbp and Mba, were identified. These conserved domain architectures of these three ORFs were further analyzed by CDART (NCBI), their subcellular localization by PSORTb [22], and their putative signal peptide sequences by SignalP [23].

**Figure 1:**
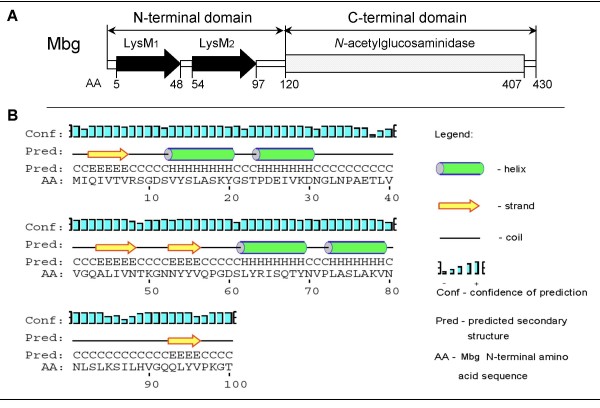
**Schematic representation of the Mbg protein**. (A) General organization of Mbg. AA, amino acid residues; LysM, lysin motif. (B) The putative secondary structure of the N-terminal domain of Mbg.

The genes *mba*, *mbp *and *mbg*, were amplified and sequenced from the *B. thuringiensis *YBT-1520 genome and their sequences were confirmed to be identical to the ORF sequence data. The gene containing two *lysM *domain *s*, *mbg*, was further characterized in terms of molecular organization and the predicted protein secondary structure. This 1290-bp gene is preceded by a putative ribosome-binding site and a promoter, as identified by -35 and -10 boxes. It encodes a protein of 430 amino acids with a deduced molecular mass of 48,112.1 Da. In structural organization, Mbg protein consists of two distinguishing domains: an N-terminal domain comprising two tandem LysM repeats (LysM_1 _from aa 5 to 48, LysM_2 _from aa 54 to 97) that was suggested to be a general peptidoglycan-binding module [14,24], and a putative C-terminal catalytic domain (Fig. 1A). There are no signal sequences found in Mbg protein. The secondary structure of the N-terminal domain was predicted by the online tool PSIPRED [25], which showed a "βααβ" fold (Fig. 1B) that coincided with the determined secondary structure of MltD, a LysM protein with peptidoglycan-binding activity in *E. coli *[24]. The amino acid sequence of the C-terminal domain of Mbg was aligned with the sequences of a spore peptidoglycan hydrolase (*N*-acetylglucosaminidase) from *B. thuringiensis *subsp. *israelensis *ATCC 35646 (GenBank: ZP_00740722). It showed 98.63% similarity in this region, suggesting that Mbg could be an *N*-acetylglucosaminidase, - a peptidoglycan-binding hydrolase.

### Expression and screening of cell wall binding protein anchors

Sequence homology analysis revealed that all three genes - *mba*, *mbp*, and *mbg *- encoded putative cell wall binding proteins. To determine their binding capacities, *gfp *was used as the reporter gene to construct the recombinant *mba-gfp*, *mbp-gfp *and *mbg-gfp*, which were assigned under the control of their endogenous promoters. The resultant transformed *B. thuringiensis *constructs, MB160, MB161, and MB162, were then analyzed for surface GFP activities. The display efficiencies of the fusion proteins in the target cells were quantified by flow cytometry.

**Figure 2:**
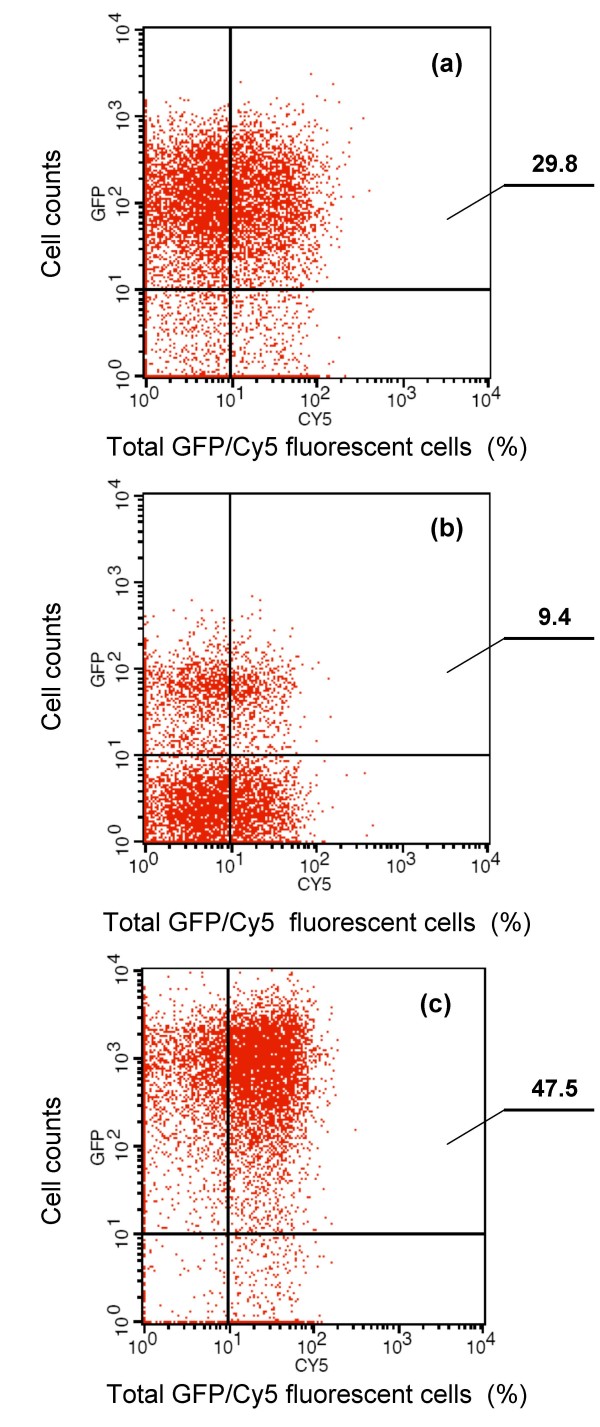
**Flow cytometry analysis of *B. thuringiensis *recombinant cells expressing (a) Mba-GFP, (b) Mbp-GFP and (c) Mbg-GFP fusion proteins**. Cells were labelled with primary monoclonal anti-GFP antibody, followed by secondary Cy5-conjugated goat anti-mouse IgG. The histogram on the top right corner of each figure shows total GFP/Cy5-labelled fluorescent cells, and the values indicate their percentages of total cell counts. For each detection, a total of 100,000 cells were analyzed.

The Mbg-associated fusion protein exhibited the highest capacity for cell surface display, as indicated by the total numbers of fluorescent Cy5-labelled cells (47.6% of total 10^5 ^determined cells) (Fig. 2), although no signal peptide sequences could be predicted for the Mbg molecule. Compared with the 668 aa Mbg-GFP, two other smaller fusion proteins - Mba-GFP consisting of 497 aa including a 32 aa signal peptide sequence and Mbp-GFP consisting of 622 aa including a 30 aa signal peptide sequence - showed only a limited cell wall binding efficiency (29.8% and 9.4% of total 10^5 ^determined cells, respectively). This feature enabled us to select Mbg as a cell-wall binding anchor for further studies.

### Verification of Mbg as a cell wall binding protein anchor

**Figure 3:**
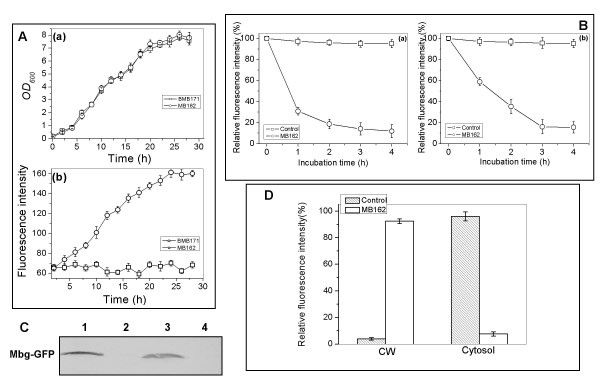
**Expression profiles of *B. thuringiensis *MB162 cells**. (A) Time course of (a) cell growth and (b) specific GFP fluorescence intensity of MB162 cells expressing Mbg-GFP fusion protein. Cells were cultured in LB at 30°C in the presence of 25 μg/mL of erythromycin, then harvested and diluted to unit cell density (OD_600 _= 1) with PBS buffer (pH7.0) for GFP fluorescence intensity determination. (B) SDS sensitivity and pronase accessibility assays of MB162 intact cells and the control cells expressing only cellular GFP. Relative values were based on GFP fluorescence intensity at the initial incubation time. Each value and error bar represents the mean of three independent experiments and its standard deviation. The values of MB162 cells without pronase or SDS treatment remained unvaried during the time course (data not shown). (C) Western blot analysis of MB162 cell fractions. Lane 1, cell-wall fraction; lane 2, soluble cytoplasmic fraction; lane 3, whole cell lysate; lane 4, BMB171 (the negative control). (D) GFP specific fluorescence intensity measurement of cell-wall (CW) and cell cytosol fractions.

The whole-cell GFP fluorescence intensity monitoring during the duration of the culture showed a steadily increasing expression pattern of fluorescence intensity with time (Fig. 3Ab), suggesting the constitutive expression of Mbg-GFP in MB162. Cell growth of MB162 was also highly consistent with that of its parent strain BMB171 (Fig. 3Aa), indicating that the added constitutive expression of the fusion protein did not create any serious burden for the host cells.

**Figure 4:**
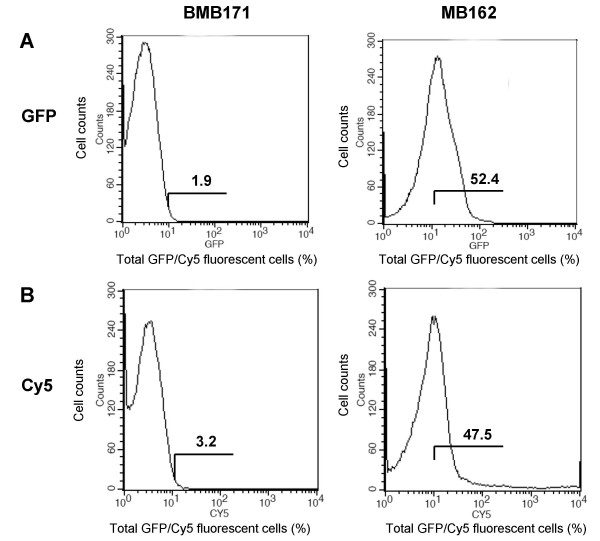
**Flow cytometric analysis of *B. thuringiensis *BMB171 and MB162 cells**. Cells were labelled with primary monoclonal anti-GFP antibody, followed by secondary Cy5-conjugated goat anti-mouse IgG. The value of each histogram indicates the percentage of total GFP/Cy5-labelled fluorescent cells. For each experiment, 100,000 cells were analyzed.

**Figure 5:**
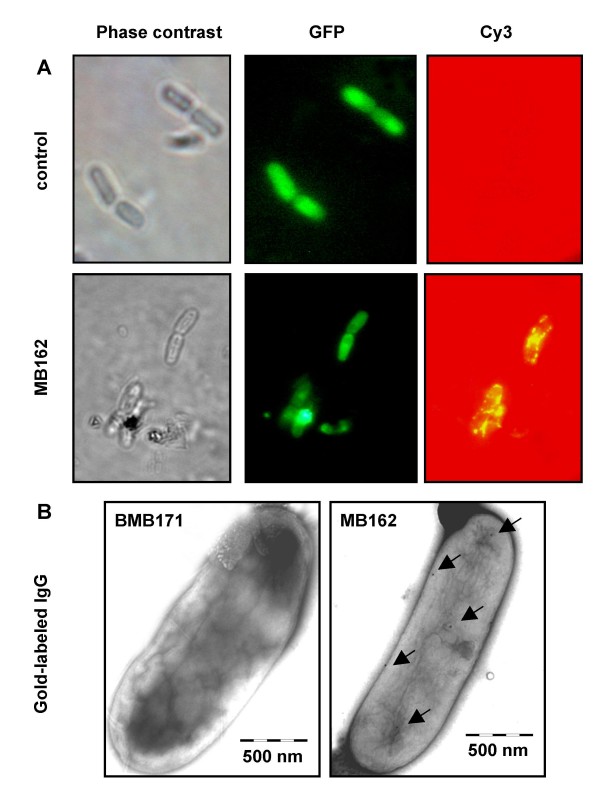
**Microscopic observation of *B. thuringiensis *MB162 cells expressing Mbg-GFP and control cells expressing cellular GFP**. For immunofluorescence microscopy (A), cells were treated with anti-GFP monoclonal antibody and followed with goat anti-mouse Cy3-conjugate antibody. Panels show the images observed by phase contrast microscopy and fluorescence microscopy using green and red emission filters. For immunochemical electron microscopy (B), the samples were further treated with 10 nm colloidal gold-conjugated goat anti-mouse IgG. Arrows indicate the gold particles. The scale bars represent 500 nm.

To confirm that Mbg-GFP fusion protein was located on the cell surface, pronase accessibility and SDS sensitivity assays (Fig. 3B) were performed. Further confirmation was obtained by western blot analysis and GFP specific fluorescence intensity determination of cell wall fractions (Fig. 3C, 3D), flow cytometry analysis (Fig. 4), immunofluorescence microscopy and TEM examination following immunogold labelling (Fig. 5).

Pronase is a specific and effective tool for detection of cell surface displayed GFP [26]. As shown in Fig. 3Ba, about 90% reduction of GFP fluorescence intensity was observed when MB162 cells were treated with pronase for 4 h, compared to only a slight reduction (less than 10%) in pronase-treated control cells (expressing intracellular GFP in the cytosol). In addition, the SDS sensitivity analysis showed a similar pattern for MB162 and control cells (Fig. 3Bb).

Subcellular fractionation was performed on equal volumes of the cell wall fractions and soluble cytoplasmic fractions. These fractions were then subjected to both Western blot analysis with an anti-GFP antibody (Fig. 3C) and GFP specific fluorescence intensity determination (Fig. 3D). Immunoblotting profiles showed that the expressed Mbg-GFP proteins were found almost exclusively in the cell wall fraction, with very little Mbg-GFP found in the cytosol fraction (a very weak band can be observed in lane 2 of Fig. 3C). This result was further confirmed by determination of the GFP fluorescence intensity of cell fractions, as shown in Fig. 3D. About 92% of the total GFP fluorescence was recovered in the cell wall fraction, whereas over 96% of total GFP fluorescence was found in the cytosol fraction of the control strain.

Flow cytometry (FACS) assays also provided evidence for surface localization. As shown in Fig. 4, while the fluorescence intensity was very weak in the control strain, a ratio of both GFP- and Cy5-associated fluorescent cells was clearly seen (52.4% for GFP and 47.5% for Cy5). This suggested that most of the expressed Mbg-GFP fusion proteins were immobilized onto the surfaces of the target cells, as shown in Figs. 3B, 3C, and 3D. However, the relatively fewer numbers of Cy5-labelled cells also reflected the low level of Mbg-GFP in MB162.

Cy3-labelled and immunogold-labelled single MB162 vegetative cells were further examined using immunofluorescence and electron microscopy. The surface displayed Mbg-GFP fusion proteins were visualized as fluorescent spots, and by the colloidal gold particles bound to the outer surface of recombinant MB162 cells (Fig. 5). These findings confirmed that Mbg-GFP fusion proteins were displayed successfully on the surfaces of target cells, and also retained their fluorescence activity. However, these results also indicated that the display efficiency of Mbg-GFP is limited.

### Effect of different Mbg domain anchors on display efficiency

**Figure 6:**
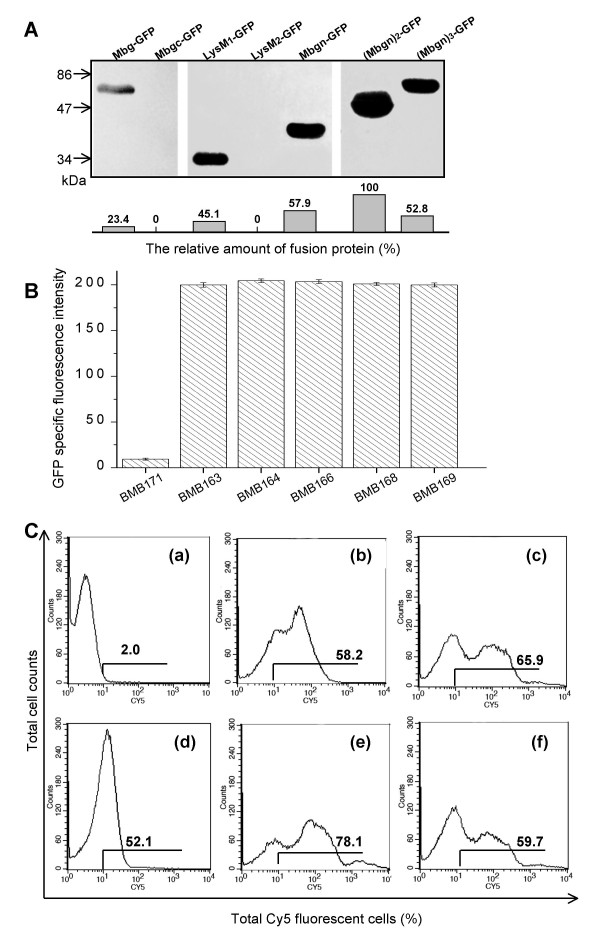
**Cell-wall binding efficiency analysis of recombinant *B. thuringiensis *cells anchored by various Mbg domains**. (A) Western blot analysis of cell-wall fractions. All fractionated samples were prepared in parallel, and an equal volume of each sample was used for analysis. The relative amount of fusion protein shown on each lane was quantified using the amount of (Mbgn)_2_-GFP as the reference value (100%). Abbreviations: Mbgn, the N-terminal domain of Mbg; Mbgc, the C-terminal domain of Mbg; lysM, the lysin motif; GFP, green fluorescence protein; (Mbgn)_2_, two tandemly aligned Mbgn repeats; (Mbgn)_3_, three tandemly aligned Mbgn repeats. (B) Total GFP fluorescence intensities of BMB171 and the recombinant *B. thuringiensis *cells expressing GFP-fusion proteins with various anchoring domains. (C) Flow cytometric analysis of BMB171 and the recombinant *B. thuringiensis *cells expressing GFP-fusion proteins with various anchoring domains. (a) BMB171; (b) MB163 cells expressing Mbg-GFP; (c) MB164 cells expressing Mbgn-GFP; (d) MB166 cells expressing LysM_1_-GFP; (e) MB168 cells expressing (Mbgn)_2_-GFP; and (f) MB169 cells expressing (Mbgn)_3_-GFP. The value of each histogram indicates the percentage of total Cy5-labelled fluorescent cells. For each experiment, 100,000 cells were analyzed.

The low display efficiency of the Mbg-GFP fusion protein on the cell surface may have been attributable to a low activity of the resident *mbg *promoter. Therefore, we constructed the recombinant pMB163 and substituted the *mbg *promoter with *P*_*cry3Aa*_, which is regarded as a weak constitutive promoter but one that is significantly expressed during the vegetative phase of growth [2]. The quantities of displayed Mbg-GFP fusion proteins in MB162 and MB163 cells determined by flow cytometry analysis showed an increase in total Cy5-labelled fluorescent cells from 47.5% to 58.2% (Fig. 6Cb and Fig. 4). This demonstrated a relatively higher activity of *P*_*cry3Aa *_for initiating Mbg-GFP expression in MB163 than that of the resident promoter of *mbg *in MB162.

**Figure 7:**
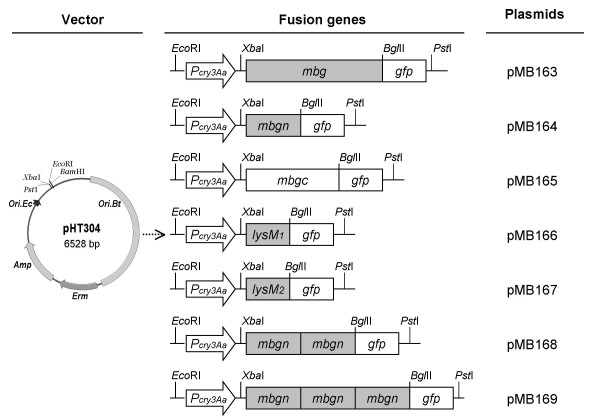
**Schematic illustration of the recombination plasmids harbouring different anchoring domains**. The resulting recombination plasmids are designated on the right array, while the middle array shows the gene maps of various domains or repeats of *mbg *that are driven by the promoter *P*_*cry3Aa*_. The region comprising cell wall binding domain LysM_1 _is highlighted. The boxes representing different domains are not shown to scale. Abbreviations: *P*_*cry3Aa*_, the promoter of *cry3Aa*; *mbgn*, the N-terminal domain of *mbg*; *mbgc*, the C-terminal domain of *mbg*; *lysM*, the lysin motif encoding domain; *gfp*, green fluorescence protein encoding gene.

Structural analysis indicated that the N-terminal domain of Mbg was responsible for peptidoglycan binding. To confirm this prediction, we constructed a series of recombinant genes by fusing different domains or repeats of *mbg *with *gfp *fragments (Fig. 7). We then used flow cytometry to compare the display efficiencies of transformed *B. thuringiensis *cells expressing these fusion genes. The intact transformed cells exhibited similar GFP fluorescence intensities (Fig. 6B). All fusion proteins harbouring the N-terminal domains exhibited cell surface localization (Fig. 6Cb, c, e, and 6f). In contrast, the sole C-terminal domain showed no cell wall binding activity (Fig.7A, lane "Mbgc-GFP"), confirming that the N-terminal domain of Mbg was the active domain for cell wall binding.

Accordingly, the fusion protein that was comprised solely of the N-terminal domain showed increased binding capacity over that of the full-length Mbg fusion protein (65.9% to 58.2%, Fig. 6Cb, c). This indicated that the smaller fusion protein was more efficiently immobilized onto the cell surface, which has also been reported by a previous investigation on the truncated ice nucleation protein of *Pseudomonas syringae *[27]. In addition, LysM_1 _associated fusion protein exhibited almost equivalent binding efficiency with that of N-terminal domain associated fusion protein (Fig. 6Cb, d). However, the LysM_2 _associated fusion protein showed no cell-wall binding signs, either by flow cytometry analysis (data not shown) or by western blot analysis of the prepared cell wall sample (Fig. 6A, lane "LysM_2_-GFP"). This again indicated that LysM_1 _acted as a functional domain for cell wall binding.

To investigate the effect of different repeats of the N-terminal domain on displaying efficiency, we constructed recombinant genes that fused two and three repeats of the N-terminal domain and the *gfp *fragment. Interestingly, the increase in anchoring motifs with three repeats of the N-terminal domain of Mbg did not elicit a coordinated increase in binding efficiency. The two repeats of the N-terminal domain associated fusion protein showed the highest binding efficiency, while the binding efficiency of three repeats of N-terminal domain was reduced (Fig. 6Ce, f). The western blot profile of all fusion proteins prepared from the cell wall fractions showed almost parallel effects to those noted with flow cytometry analysis, as shown in Fig. 6A. Thus, we affirmed that the two tandem repeats of the N-terminal domain of Mbg could serve as an optimum anchoring motif for efficient protein display on the surface of *B. thuringiensis *BMB171 cells.

### Cell surface display of bacterial laccase on *B. thuringiensis *BMB171

**Figure 8:**
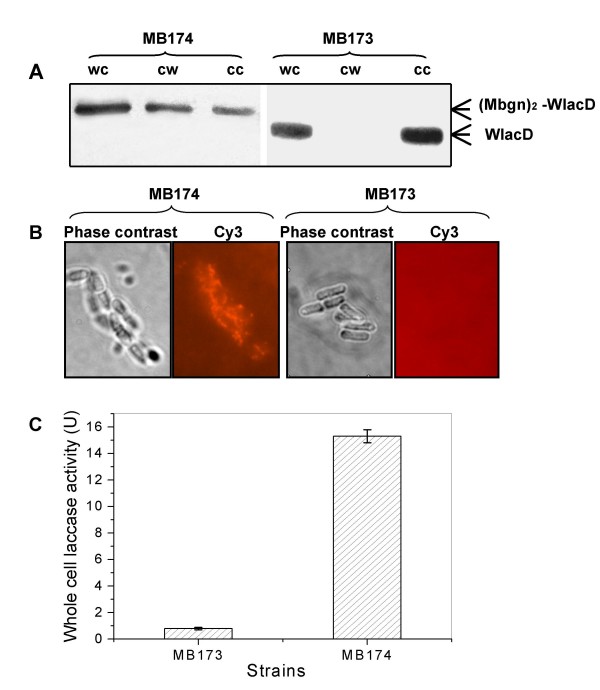
**Verification of surface display of WlacD in recombinant *B. thuringiensis *MB174 cells**. (A) Western blot analysis of cell fractions prepared from MB174 cells expressing (Mbgn)_2_-wlacD and MB173 cells expressing cellular wlacD. WC, whole cell fractions; CW, cell-wall fractions; CC, cell cytoplasmic fraction. (B) Microscopic observation of MB173 and MB174 intact cells that were treated with anti-WlacD polyclonal antibody and followed with goat anti-mouse Cy3-conjugate antibody. (C) Measurement of whole-cell laccase activity using ABTS as the substrate. Each value and error bar represents the mean of three independent experiments and its standard deviation.

A previously mutated bacterial laccase (WlacD, 523 aa), has shown high catalytic activity even as part of a fusion pattern [20], so we chose this enzyme as a fusion partner to develop a whole-cell catalyst. For this purpose, the encoding gene of laccase, *wlacD*, was positioned in the frame of two tandem repeats of the *mbgn *gene, to generate a recombinant pMB174 expressing the fusion gene of (*mbgn*)_2_-*wlacD*. The Western blot and immunofluorescence microscopy profiles showed clear signs for cell wall binding in the recombinant MB174 cells, but not for the MB173 cells that expressed the cellular WlacD (Fig. 8A, B). However, unlike the pattern using GFP as the partner, a thick band for the cell cytosol sample (Fig. 8A, lane "CC" for MB174) indicated that cell expressed recombinant enzyme molecules in MB174 cells had been only partially carried to the cell surface. Nevertheless, the laccase activity measurement showed that the whole-cell enzymatic activity was 15.3 U per/ml (strain concentration OD_600 _= 1.0), whereas only slight activity was found for the MB173 cells used as the negative control (Fig. 8C).

## Discussion

The aim of this study was to establish a novel cell surface display system that would enable the display of target proteins. *B. thuringiensis *appears to be particularly valuable for this purpose because of its rigid cell-wall structure and distinctive biosynthesis capability. The LysM domain is one of most common modules in bacterial cell surface proteins [14]. Increasingly more bacterial genomic sequences are being released that are providing effective and rapid approaches to recognizing these LysM-containing proteins by sequence homology analyses. In the present study, the *N*-acetylglucosaminidase (Mbg) we employed has been proven to have the ability to target different heterologous proteins (GFP or WlacD) onto the surface of the host cells, raising the possibility of cell surface display of other proteins, such as antimicrobial peptides/proteins, protective proteins, or quorum sensing signal molecules, onto *B. thuringiensis *cells using Mbg as the anchoring motif. This strategy would have practical applications for the development of agricultural bioinsecticides that could have additional antimicrobial activity, or could have increased persistence in the field.

The cell-wall binding assay demonstrated that Mbg was primarily bound to the surface of the target cells, which meant that it must have been secreted across the cell membrane. Deletion of the C-terminal domain did not attenuate the binding efficiency, and the intramolecular truncated LysM_1 _showed a comparable binding efficiency with the N-terminal domain (Fig. 6A, C). These results suggested that Mbg must be exported to the cell wall via some other signal pathway that was peptide-independent.

In *B. subtilis*, certain extracellular proteins are secreted by the Sec-SRP pathway, or Twin-arginine translocation pathway, or ATP-binding cassette (ABC) transporter channel [28,29]. The transmembrane localization and secretion of these proteins are largely attributed to their cleavable signal peptides; however, the route for Mbg-like proteins that lack transmembrane segment (TM) and signal peptide is currently unknown. In fact, several other Mbg-like anchor proteins, such as CwlC of *B. subtilis *[30], CwlM of *B. licheniformis *[31], Endo-BH, -BL and -LP of *B. halodurans *[32], and a major secretory protein of Gram-negative bacteria *P. syringae*, ice nucleation protein (INP), also lack signal peptide sequences [33]. It therefore remains a challenge to elucidate the mechanism of Mbg-like anchor proteins for transport across the parent cell membrane.

For a developing cell surface display system, it is desirable to obtain anchor proteins that are endowed with a high capacity for cell surface binding. For this purpose, we initially substituted the resident promoter of *mbg *with *P*_*cry3Aa*_, a sporulation-independent weak promoter, but one that could be activated during the vegetative growth [2]. This resulted in elevated expression levels, but in some cases, the high-level expression did not result in a matching cell surface display. For instance, in the case of INP-associated GFP display, only a small amount of anchor fusion bound to the membrane - most of it was retained in the cytoplasm [27]. This did not occur with the constitutive promoter of Mbg (*P*_*mbg*_) or with *P*_*cry3Aa *_driven Mbg-GFP expression. In all cases, the majority of Mbg-GFPs were translocated to the surface of the target cells, whereas only a small amount remained in the cytoplasm (Fig. 3, 7A). This indicated that under weak transcription activity, cellular transcription and secretion would be coordinated, thus allowing efficient display of GFP proteins. However, when the larger laccase was fused to two tandemly aligned N-terminal domains of Mbg, a considerable amount of fusion protein was found in the cytoplasm (Fig. 8A).

Several previous studies have reported on strategies to improve the display capacity of living cells by increasing the number of anchor proteins [15,34,35]. To investigate if this strategy could be applied to the Mbg-associated display system, we constructed two and three tandemly aligned repeats of N-terminal domain, which were fused with the N-terminus of GFP to allow expression on the *B. thuringiensis *cell surface. As shown in Fig. 6A and 6C, a domain-increased anchoring motif with two tandemly aligned repeats exhibited the highest display efficiency (by 78.1%). This contrasted with 58.2% of this activity for the full-length Mbg anchor, and 65.9% for sole N-terminal domain anchor.

Surprisingly, when the anchor numbers were increased to three tandem repeats of the N-terminal domain, a significant reduction in display efficiency occurred in comparison with that of either of the two repeats or the sole N-terminal anchor (Fig. 6C). Our current hypothesis to explain this result is that the number of residues in the fusion protein is essential for translocation. The Mbg-associated fusion protein must not exceed a critical length of approx. 450 amino acids (close to 430 aa of Mbg) to allow for efficient transport. This is probably a requirement to prevent the length of the fusion protein from exceeding the actual length of Mbg (430 aa). While the (Mbgn)_2_-GFP fusion was substantially displayed on the cell surface, as a result of being well matched to Mbg in length (by 438 aa), it is conceivable that the elongated (Mbgn)_3_-GFP (by 528 aa) and (Mbgn)_2_-WlacD (by 723 aa) exhibited a reduction in binding efficiencies.

A mutated bacterial multicopper oxidase (laccase) was immobilized in an active conformation onto the cell wall of *B. thuringiensis*, resulting in a whole-cell catalyst. While most of the displayed enzymes currently tend to be hydrolases, the result of this study demonstrated that it is possible to surface display an oxidase without radically altering its catalytic activity. This is the first report of successful display of an oxidase in a *B. thuringiensis *system using the LysM anchor. Owing to their capacity for substantial non-specific oxidation of numerous substrate types (include a variety of environmentally hazardous chemicals), laccases have a number of important commercial uses, including decolorization of textile dyes, bioremediation, effluent treatment, and other industrial processing. The demonstration of cell surface display and activity in this study points to a valuable practical application of this novel cell surface technology in the form of regenerative and flow-through enzymatic systems for use in bioconversions.

## Conclusion

We constructed and compared the ability of a new peptidoglycan hydrolase of *B. thuringiensis *to direct cell surface display of two target foreign proteins, GFP and bacterial multicopper oxidase (WlacD). The N-terminal domain of Mbg contributed to cell-wall anchoring, while LysM_1 _was the active domain. Two tandemly repeated Mbgn domains exhibited the highest display activity, while the activity of three Mbgn repeats was decreased, indicating that the length of the fusion protein determined transmembrane transport. The strategy presented here could be applied to cell surface display of other heterologous proteins in *B. thuringiensis*. Moreover, since the LysM domain of *N*-acetylglucosaminidase appears to be universal to Gram-positive bacteria, it could be used to develop similar systems in many other bacteria.

## Methods

### Bacterial strains and culture conditions

*B. thuringiensis *subsp. *kurstaki *wild-type strain YBT-1520 provided the gene resources for screening autolysin genes. A plasmid-free derivative of *B. thuringiensis *BMB171 was used as the host strain for surface display of recombinant proteins. Competent *E. coli *DH5α cells (TaKaRa Bio Inc.) were used to construct various recombinant plasmids. Transformed *B. thuringiensis *strains harbouring recombinant plasmids were grown at 30°C in Luria-Bertani (LB) medium containing 25 μg/ml (w/v) of erythromycin. Transformed *E. coli *DH5α cells were grown at 37°C in LB medium supplemented with 100 μg/ml (w/v) of ampicillin.

### Plasmid construction and transformation

The oligonucleotide primers and plasmids used in this study are listed in Table 1. Three putative cell-wall binding protein encoding genes, *mba*, *mbp *and *mbg*, together with their promoter sequences, were amplified by PCR with primers BT101/BT102, BT301/BT302 and P1/P2, respectively, from the *B. thuringiensis *YBT-1520 genome. To construct the fusion gene *mba-gfp*, the green fluorescence protein gene (*gfp*) was amplified with primers BT103 and P4 from plasmid pGFPuv (Clontech). The fused *mba-gfp *was generated by PCR with primers BT101 and P4, using the mixed *mba *and *gfp *fragments as the DNA template by following the standard SOE (splicing by overlap extension) method [36]. The resulting fragment was digested with *Eco*RI and *Pst*I and then inserted into *Eco*RI/*Pst*I sites of *E. coli*-*B. thuringiensis *shuttle vector pHT304, resulting in plasmid pMB160. Similar SOE methods were performed to construct recombinant pMB161 which harboured the *mbp-gfp *fusion gene and pMB162 harbouring the *mbg-gfp *fusion gene. Primers BT303/P4 were used to amplify *gfp *and BT301/P4 were used to amplify *mbp-gfp *for constructing pMB161. Primers P3/P4 were used to amplify *gfp*, P1/P4 were used to amplify *mbg-gfp*, and *Sma*I/*Pst*I were used to construct pMB162.

**Table 1:** Plasmids and oligonucleotide primers used in this study

**Plasmids or primers**	**Phenotypes or sequences**^*a*^	**Source or reference**
Plasmids		
pHT304	Amp^r^Em^r^, *E. coli*-*B. thuringiensis *shuttle cloning vector, 6528 bp	[39]
pGFPuv	Amp^r^, plasmid vector carrying a variant of *gfp*, 3336 bp	CLONTECH Lab, Inc.
pBMB3305	Amp^r^Em^r^, pHT304 derivative harbouring insecticidal gene *cry3Aa *with promoter *P*_*cry3Aa*_, ~12.6 kb	Laboratory collection
pMB172	Amp^r^, pTYB12 derivative harbouring *wlacD*, 8867 bp	[20]
pMB160	Amp^r^Em^r^, pHT304 derivative harbouring *mba-gfp *fusion gene, 8194 bp	This study
pMB161	Amp^r^Em^r^, pHT304 derivative harbouring *mbp-gfp *fusion gene, 8667 bp	This study
pMB162	Amp^r^Em^r^, pHT304 derivative harbouring *mbg-gfp *fusion gene, 8648 bp	This study
pMB163	Amp^r^Em^r^, pHT304 derivative harbouring promoter *P*_*cry3Aa*_, *mbg *and *gfp*, 9365 bp	This study
pMB164	Amp^r^Em^r^, pHT304 derivative harbouring *P*_*cry3Aa*_, *mbgn *and *gfp*, 8466 bp	This study
pMB165	Amp^r^Erm^r^, pHT304 derivative harbouring *P*_*cry3Aa*_, *mbgc *and *gfp*, 9002 bp	This study
pMB166	Amp^r^Em^r^, pHT304 derivative harbouring *P*_*cry3Aa*_, *lysM*_1 _and *gfp*, 8276 bp	This study
pMB167	Amp^r^Em^r^, pHT304 derivative harbouring *P*_*cry3Aa*_, *lysM*_2 _and *gfp*, 8294 bp	This study
pMB168	Amp^r^Em^r^, pHT304 derivative harbouring *P*_*cry3Aa*_, (*mbgn*)_2 _and *gfp*, 8777 bp	This study
pMB169	Amp^r^Em^r^, pHT304 derivative harbouring *P*_*cry3Aa*_, (*mbgn*)_3 _and *gfp*, 9098 bp	This study
pMB173	Amp^r^Em^r^, pHT304 derivative harbouring *P*_*cry3Aa *_and *wlacD*, 8879 bp	This study
pMB174	Amp^r^Em^r^, pHT304 derivative harbouring *P*_*cry3Aa*_, (*mbgn*) _2 _and *wlacD*, 9521 bp	This study
Oligonucleotide primers^b^		
BT101	5'-CGAGAATTCTGACAAGTGCGACACGTT-3'	
BT102	5'-TTCTTCTCCTTTACTCATACAGAAAATATGTTTACCG-3'	
BT103	5'-CGGTAAACATATTTTCTGTATGAGTAAAGGAGAAGAA-3'	
BT301	5'-CGTGAATTCCACTGTCAGTATAACACC-3'	
BT302	5'-TTCTTCTCCTTTACTCATCCTAACTAAATATGGCAG-3'	
BT303	5'-CTGCCATATTTAGTTAGGATGAGTAAAGGAGAAGAA-3'	
P1	5'-TTACCCGGGCTTCCCTTCTTTCACTTC-3'	
P2	5'-TTCTTCTCCTTTACTCATGCCCTTTTTCGTAATCGT-3'	
P3	5'-ACGATTACGAAAAAGGGCATGAGTAAAGGAGAAGAA-3'	
P4	5'-AAACTGCAGTTATTTGTAGAGCTCATCCATGC-3'	
P5	5'-ACGGGAATTCGGATTCAAAATAGCCCTG-3'	
P6	5'-CTGTCTAGACGGATTCATTTTTCTTCC-3'	
P7	5'-CCAAGTTCTAGAATGATTCAAATTGTAACGG-3'	
P8	5'-GCTAGATCTGCCCTTTTTCGTAATCGT-3'	
P9	5'-ACGAGATCTATGAGTAAAGGAGAAGAA-3'	
P10	5'-TCTAGAGATCTGATGGATTCTACAGCTCG-3'	
P11	5'-CTGTCTAGAGTTAATGCTACACGTGCC-3'	
P12	5'-GCAAGATCTAACGATAAGTGCCTGACC-3'	
P13	5'-CGCTCTAGATATGTACAGCCTGGTGAC-3'	
P14	5'-CGCGGATCCATGATTCAAATTGTAACGG-3'	
P15	5'-CTGTCTAGA ATGCAACGTCGTGATTTC-3'	
P16	5'-AAACTGCAGTTATACCGTAAACCCTAAC-3'	
P17	5'-GCAAGATCTATGCAACGTCGTGATTTC-3'	

^a ^Em^r^, erythromycin resistance; Amp^r^, ampicillin resistance; *cry3Aa*, *B. thuringiensis *insecticidal gene;*P*_*cry3Aa*_, the promoter of *cry3Aa*; *gfp*, green fluorescent protein gene; *wlacD*, a mutated *S. dysenteriae *laccase gene [20]; *mbg*, a putative *N*-acetylglucosaminidase gene from *B. thuringiensis *YBT-1520; *mbgn*, N-terminal domain of *mbg*; *mbgc*, C-terminal domain of *mbg*; (*mbgn*)_2_, two tandemly aligned repeats of N-terminal domain of *mbg*.

^b ^The underlined sequences indicates the restriction enzyme sites.

The fusion genes were sequenced to verify the correctness of their sequences. The *mbg-gfp *fusion gene was under the control of a sporulation-independent promoter *P*_*cry3Aa*_, which could be activated during vegetative growth [2]. The sequence of promoter *P*_*cry3Aa *_was amplified by PCR using primers P5 and P6 and then digested with *Eco*RI and *Xba*I. The encoding sequence of *mbg *(without terminator sequence) was amplified using primers P7 and P8 and digested with *Xba*I and *Bgl*II. The encoding sequence of *gfp *was amplified using primers P9 and P4 and digested with *Bgl*II and *Pst*I. These three digested fragments were ligated sequentially into one fragment, *in vitro*, and then inserted into *Eco*RI and *Pst*I sites of pHT304. The resulting plasmid was designated pMB163.

This plasmid was further used to construct several recombinant genes fused with different domains of *mbg *and *gfp*. Among these, the N-terminal domain of *mbg *(*mbgn*) was amplified using primers P7 and P11, then digested with *Xba*I and *Bgl*II and inserted into the *Xba*I/*Bgl*II sites of pMB163 to give plasmid pMB164. The C-terminal domain of *mbg *(*mbgc*) was amplified using primers P11 and P8, LysM_1 _was amplified using primers P7 and P12, LysM_2 _was amplified using primers P13 and P10, and these amplified fragments were digested and ligated similar to the procedure for pMB164, to create plasmids pMB165, pMB166 and pMB167, respectively.

To construct recombinant plasmids expressing two or three repeats of *mbgn*, PCR amplification of *mbgn *was performed using primers P14 and P10, then digested with *Bam*HI and *Bgl*II and inserted into the *Bgl*II site of pMB164. The resulting plasmid was verified by restriction enzyme digestion analysis, which harboured two repeats of *mbgn *at the same orientation, and was designated pMB168. A further similar insertion of the above digestion fragment into the *Bgl*II site of pMB168, and verification of construction, was performed to give plasmid pMB169, which harboured three repeats of *mbgn *at the same orientation.

To construct a recombinant gene expressing the bacterial laccase, WlacD, PCR was performed to amplify *wlacD *using the primers P15 and P16, with plasmid pMB172 as DNA template, followed by digestion with *Xba*I and *Pst*I and insertion into *Xba*I/*Pst*I sites of pMB163. This resulted in pMB173. To construct a (*mbgn*)_2_-*walD *fusion gene, *wlacD *was amplified by PCR using primers P17 and P16, then digested with *Xba*I and *Pst*I and ligated to the same sites of pMB168. The resultant plasmid was designated pMB174. All of the PCR products and insertion fragments were confirmed by sequencing.

Recombinant plasmids were transformed into *E. coli *by standard procedures [36]. Transformation of *B. thuringiensis *by electroporation was performed as described previously [20]. Transformed *B. thuringiensis *BMB171 strains harbouring plasmid pMB160, pMB161, pMB162, pMB163, pMB164, pMB165, pMB166, pMB167, pMB168, pMB169, pMB173 and pMB174, were designated as MB160, MB161, MB162, MB163, MB164, MB165, MB166, MB167, MB168, MB169, MB173 and MB174, respectively.

### Fluorescence assays

Cell density was measured at 600 nm with a UV/VIS spectrophotometer (DU-800 Nucleic Acids/Protein Analyzer, Beckman Coulter). Prior to the GFP fluorescence intensity determination, cells were harvested and diluted to unit cell density (OD_600 _= 1.0) with PBS buffer (pH7.0), with similarly diluted *B. thuringiensis *BMB171 cells used as a background reference. Specific GFP fluorescence intensities of whole-cell and/or cell fractions were determined using a fluorescence spectrophotometer (RF-5103PC, Shimadzu, Japan) at an excitation of 495 nm and emission of 509 nm.

### Cell fractionation and preparation of cell surface proteins

A 100 ml volume of recombinant strain was grown at 28°C for 24 hours, then harvested and washed three times with PBS buffer (pH7.0). The cell suspension was passed twice through a French Pressure Cell (Thermo, USA) at 20,000 psi. The suspension was then fractionated following the method described previously for *B. subtilis *[30]. Equal volumes of each fractionated sample were used for GFP fluorescence intensity determination and Western blot analysis.

Cell surface proteins were prepared essentially using the 5 M LiCl method described by Kuroda and Sekiguchi [37]. Fractions containing cell surface proteins were ultimately precipitated by adding 2% trichloroacetic acid, letting the solution stand for 30 min, then centrifuging at 20,000 × *g *for 5 min. The resulting pellets were solubilized in a sample buffer for SDS-polyacrylamide gel electrophoresis (SDS-PAGE).

### Western blot analysis

SDS-PAGE was performed as described by Laemmli [38]. Western blot analysis was performed as described by Li et al. [27]. The relative amounts of proteins in western blot images were analyzed by Quantity One 1-D Analysis Software (Bio-Rad).

### Protease accessibility and SDS sensitivity assays

For pronase accessibility assays, cells were harvested and washed three times with PBS, then adjusted to OD_600 _of 1.0. Pronase (4.7 Unit/mg, Sigma) was added to a final concentration of 0.5 mg/ml. Cell suspensions were incubated at 37°C. SDS was also added to each cell suspension to a final concentration of 0.05% (wt/vol). The GFP fluorescence intensity of Pronase- and SDS-treated cell suspensions and control were measured at hourly intervals.

### Immunofluorescence microscopy and immunogold electron microscopy

The harvested cells were washed three times with PBS buffer (pH 7.2), suspended in PBS buffer with 1% BSA containing anti-GFP monoclonal antibody (Chemicon) at a 1:200 dilution, and incubated at room temperature for 2 h. After washing three times with PBS, the samples were resuspended in 1% BSA-PBS buffer containing goat anti-mouse Cy3-conjugate antibody (Invitrogen, Carlsbad, CA) and further incubated at room temperature for 2 h. The cells were washed three times and examined by optical-fluorescent phase-contrast microscopy (Olympus BX51).

Immunogold-labelling for electron microscopic observation was performed by fixing samples for 30 min in 2% glutaraldehyde at room temperature, washing, then incubating in anti-GFP monoclonal antibody (Chemicon) buffer for 1 h. The samples were then resuspended in 1% BSA-PBS buffer containing 10 nm colloidal gold-conjugated goat anti-mouse IgG (Sigma) at 1:100 dilution, further incubated for 2 h at room temperature, then picked onto Formvar carbon-coated copper grids, post-stained with 2% aqueous uranyl acetate, air dried, and examined under a TECNAI G^2 ^transmission electron microscope (FEI) at 200 kV.

### FACS analysis

Flow cytometry analysis was performed to confirm the surface localization of the recombinant GFP fusion proteins. An overnight culture of BMB171 (as the negative control) and the recombinant cells were harvested and washed three times with PBS, then resuspended in PBS containing 1% skim milk and monoclonal anti-GFP antibody (1:200), and incubated on ice for 1.5 h. After washing three times with PBS, the cells were incubated with Cy5-conjugated goat anti-mouse IgG (1:100, v/v) (Invitrogen, Carlsbad, CA) on ice for 1 h. The Cy5-labeled cells were examined using a FACScan flow cytometer (Becton Dickinson, Oxnard, CA) equipped with a dual laser system (Ar: 488 nm and He-Ne: 633 nm) for simultaneous measurement of GFP and Cy5 fluorescence (Cy5 could be excited by only 63% of total fluorescence). The results were expressed as the percentage of total GFP/Cy5-labelled fluorescent cells (defined as the value of the fluorescence intensity ≥ 10). For each experiment, 100,000 cells were analyzed.

### Laccase antiserum preparation, immunofluorescence microscopy and western blot analysis

A mutated laccase WlacD from *S. dysenteriae *has been previously expressed and purified in *E. coli *system [20]. To prepare polyclonal laccase antiserum, 500 μg of the purified WlacD protein was subcutaneously injected into the neck region of a New Zealand rabbit after being emulsified with Freund's Complete adjuvant. A total of four subsequent booster injections were performed at days 14, 21, 28 and 35. The antiserum was collected 10 days after the last injection, and was centrifuged at 4000 × *g *for 10 min at 4°C. NaN_3 _at a final concentration of 0.1% was added to the supernatant, which was then stored in -80°C until use. Immunofluorescence microscopy and Western blot analyses of the recombinant *B. thuringiensis *cells expressing WlacD-fusion proteins was essentially performed as described above, except that the anti-WlacD polyclonal antiserum (1: 1,000) was used as the primary antibody.

### Measurement of whole cell laccase activity

Measurement of laccase activity was performed as described previously [20]. One unit of enzyme activity was defined as the amount oxidizing 1 μmol of 2,2'-azino-bis (3-ethyl-benzthiazoline-6-sulfonic acid) (ABTS) (Amresco) per minute. Each assay was performed at least in triplicate.

### Statistical analysis

All data were averaged from triplicate assays. Statistical analysis was carried out using SPSS 13.0 statistical software. Statistical significance was defined as *P *< 0.05.

## Authors' contributions

XS performed most of the experiments, made most of the data evaluation and interpretation, drafted parts of the manuscript. MJ participated in gene cloning and plasmid construction. ZY provided the genome data of *B. thuringiensis *wild-type strain YBT-1520 and participated in the design of the study. HC prepared anti-WlacD antiserum. LL was the primary responsible for the whole work, conceived and directed the study, and revised the manuscript. All authors read and approved the final manuscript.
